# Redox-Active Ferrocene grafted on H-Terminated Si(111): Electrochemical Characterization of the Charge Transport Mechanism and Dynamics

**DOI:** 10.1038/s41598-019-45448-w

**Published:** 2019-06-19

**Authors:** Claudio Fontanesi, Enrico Da Como, Davide Vanossi, Monica Montecchi, Maria Cannio, Prakash Chandra Mondal, Walter Giurlani, Massimo Innocenti, Luca Pasquali

**Affiliations:** 1 DIEF, University of Modena and Reggio Emilia, via Vivarelli 10, 41125 Modena, Italy; 2 Department of Physics, University of Bath, Claverton Down, Bath, BA2 7AY United Kingdom; 3 DSCG, University of Modena and Reggio Emilia, via Campi 183, 41125 Modena, Italy; 4 Department of Chemistry, Indian Institute of Technology, Kanpur, 208016 India; 5 Department of Chemistry, University of Firenze, via della Lastruccia 3, 50019 Sesto Fiorentino, FI Italy; 6 IOM-CNR Institute, Area Science Park, SS 14 Km, 163.5, Basovizza, 34149 Trieste Italy; 7 Department of Physics, University of Johannesburg, P.O. Box 524, Auckland, Park 2006 South Africa

**Keywords:** Chemistry, Materials for devices

## Abstract

Electroactive self-assembled monolayers (SAMs) bearing a ferrocene (Fc) redox couple were chemically assembled on H-terminated semiconducting degenerate-doped *n*-type Si(111) substrate. This allows to create a Si(111)|organic-spacer|Fc hybrid interface, where the ferrocene moiety is covalently immobilized on the silicon, *via* two alkyl molecular spacers of different length. Organic monolayer formation was probed by Laser Ablation-Inductively Coupled Plasma-Mass Spectrometry (LA-ICP-MS) and X-ray photoelectron spectroscopy (XPS) measurements, which were also used to estimate thickness and surface assembled monolayer (SAM) surface coverage. Atomic force microscopy (AFM) measurements allowed to ascertain surface morphology and roughness. The single electron transfer process, between the ferrocene redox probe and the Si electrode surface, was probed by cyclic voltammetry (CV) measurements. CVs recorded at different scan rates, in the 10 to 500 mV s^−1^ range, allowed to determine peak-to-peak separation, half-wave potential, and charge-transfer rate constant (*K*_*ET*_). The experimental findings suggest that the electron transfer is a one electron quasi-reversible process. The present demonstration of surface engineering of functional redox-active organometallic molecule can be efficient in the field of molecular electronics, surface-base redox chemistry, opto-electronic applications.

## Introduction

**Figure 1:**
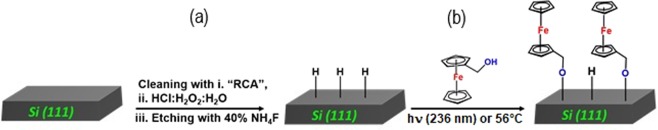
Illustration of the surface modification of the H-terminated silicon substrates. The process involves two steps. Step (**a**): (i) cleaning the silicon (111) substrates with RCA, and HCl:H_2_O:H_2_O (iii) etching with 40% NH_4_F (pH = 2, obtained by addition of few drops of concentrated sulfuric acid) to achieve H-terminated silicon. Step (**b**) covalent grafting of hydroxymethyl ferrocene on H-terminated silicon.

Over the past decades, surface-confined nanometric electroactive molecular assemblies have been the subject of key research, which play a major role in understanding redox processes^1–7^. Electroactive materials offer several advantages which include molecular structure-electronic properties correlation and tunability^8–15^. In particular, silicon based hybrid systems are of paramount interest allowing to combine the typical semiconducting properties of Si and the flexible functionalities proper of the organic materials^16–19^. Successful designed molecular architectures produce surface-based sensors, catalytic active surfaces, molecular devices, energy storage, to name a few. Among the redox-active moieties, ferrocene-based derivatives form a class of attractive organic compounds, which can be considered as model systems. This is due to ferrocene structural stability, aromaticity, ease of modification, associated to a reversible single electron transfer process (Fc^+^/Fc), low oxidation/reduction potential, and easy to prepare self-assembled monolayers (SAMs)^20,21^. In view of the above fascinating behaviour, ferrocene based SAMs could be exploited as memory elements, where the ferrocene redox centre is used as the charge storage unit and considering the molecule in the neutral or oxidized form as the two states of a bit^22^. This kind of supramolecular architectures were experimentally characterized in an extensive way as far as concerns the electron transfer ET process^19,21,23–27^, while from a theoretical point of view the fundamental knowledge of the ET dynamics, at an hybrid interface, is a subject yet open for discussion^20,28,29^. Indeed, most of the studies so far refer to ferrocene-terminated alkane thiols attached to a gold substrate. For example, a recent study by Wong *et al*. show that Fc-alkanethiol SAMs form strong ion pairs in the oxidized state which increase the chain length by more than 1 nm as probed by combining electrochemical and XPS measurements^30^. However, techniques to assemble the ferrocene derivative on the different substrates may vary depending on the functional group available. Thus, the functionalization of a molecule-electrode for studying interfacial properties needs a special attention. Different fruitful methodologies exist for the creation of hybrid interfaces, relying on the covalent grafting of functional organic molecules, organometallics, inorganic complexes and these are generally based on ultra-high vacuum (UHV) depositions^31–34^, wet chemistry^1,19^, electrochemical based methodologies^35^. Fc-SAMs other than the thiolated groups, especially Fc containing alcohol groups are basically unexplored. In this paper, we focus on silicon-based interfaces, as silicon is a linchpin material for micro-electronic industries and can be chemically functionalized by covalent grafting of the ferrocene derivatives. The wet chemistry method is the most widely used technique, due to the simple lab procedures and the vast number of reactive groups that can be grafted on the varied substrates. Herein, we study laboratory synthesized ferrocene derivatives covalently grafted on freshly prepared H-terminated silicon (111) substrate (Fig. 1).

**Figure 2:**
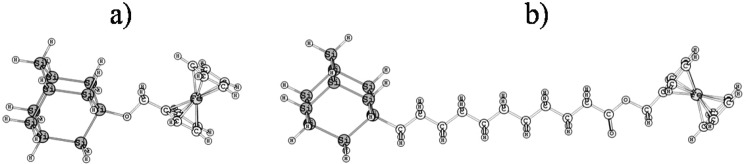
Schematic representation of the molecular architectures grafted on the Si(111) surface (the silicon surface is approximated as a cluster of ten silicon atoms whose dangling valences are saturated by hydrogens): (**a**) Si−Me−Fc (**b**) Si−UA−Fc. The relevant cartesian coordinates, are available in the Supporting Information^36^.

This study is carried out by varying the length of the chain (spacer) linking the ferrocene moiety to the surface: (1) methanol-ferrocene directly grafted on the surface (2) ferrocene grafted through a 1-iodoundecanoic acid (UA) monolayer, Fig. 2.

Formation, surface coverage, elemental analysis, electron-transfer kinetics of the Fc-SAMs on H-terminated silicon was ensured by X-ray Photoelectron Spectroscopy (XPS), Laser Ablation-Inductively Coupled Plasma-Mass Spectrometry (LA-ICP-MS), cyclic voltammetry (CV) measurements. The experimental outcome is compared to theoretical results concerning the dynamics of the electron transfer as obtained by application of the Marcus theory^36^. From the theoretical point of view, our main goal is to verify if Marcus theory^37^ is correctly capable in reproducing ET rate constants obtained experimentally. Note that, some recent works^20,28^ were devoted to point out that the Marcus model, which describes the ET dynamics in the incoherent limit^38^, fails to give a correct prediction of the ET rate constants. Particular attention is also devoted to draw a parallelism between the striking differences observed for the ET dynamics, probed by CV measurements, in the case of ferrocene covalently immobilized, or in bulk solution, in the case of SAMs of comparable thickness.

## Results and Discussion

### XPS analysis

**Figure 3:**
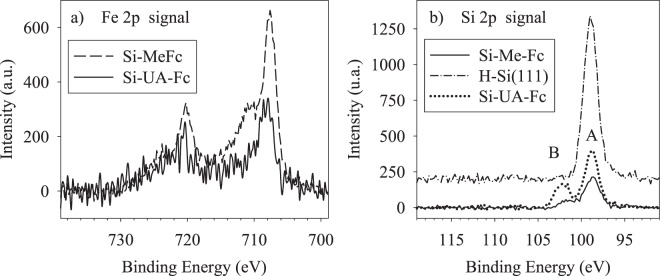
(**a**) Fe 2p XPS signal for Si−Me−Fc (continuous line) and Si−UA−Fc (dotted line) surfaces. (**b**) Si 2p levels for the pristine surface (broken-dotted line) and for Si−Me−Fc (continuous line) and Si−UA−Fc (dotted line) surfaces.

XPS measurements were performed on the freshly prepared Si−Me−Fc and Si−UA−Fc surfaces. Figure 3a sets out the details of the XPS signal in the 690–740 eV range, the region of Fe 2p binding energy. The spectra show prominent features at 707.8 eV and 721 eV, which are associated with Fe 2p_3/2,_ 2p_1/2_, respectively of Fc-CH_2_-OH deposited on H-Si(111)^39–41^. Experimental data show a spin orbit splitting at 13.2 eV and a similar spin-orbit splitting was reported with other Fe^II^-polypyridyl complexes^39^. The broad shoulder at about 711 eV is instead related to the presence of some amount of ferrocenium, which has been typically observed for these systems, either due to the manipulation of the samples or to the irradiation of the films under the X-ray beam^39–41^. A shake-up satellite contributes to the high binding energy tail of Fe 2p_3/2_, at about 714 eV^39–41^. In Fig. 3b the Si 2p is shown. The pristine surface presents a single peaked structure centred at 99 eV, indicating no traces of surface oxidation^42,43^. Indeed, a non-negligible amount of oxidised Si is found on the Si−UA−Fc surface. To assess quantitatively the Si(111) surface coverage, the attenuation of the Si 2p signal from the substrate was considered, assuming an inelastic mean free path (IMFP), λ, of 31 Å (at the kinetic energy of the Si photoelectrons of 1150 eV) in the organic layer^44^. The film effective thickness was derived applying the standard formula $d=\lambda \,\cos \,\theta \,\mathrm{ln}({I}_{Si2p,0}/{I}_{Si2p,d})$, where θ = 44.1° is the average emission angle and ${I}_{Si2p,0(d)}$ are the peak intensities of the Si2p levels from the bare substrate and from the covered sample with a film of effective thickness d, respectively^45^. We obtained an effective thickness, d = 32 Å (with a tilt angle of about 80 degrees with respect to the silicon surface) and 16 Å (with a tilt angle of about 68 degrees with respect to the silicon surface) for the Si−Me−Fc and Si−UA−Fc surfaces, respectively. This result may suggest, by comparison with optimized structures and relevant geometrical parameters obtained by DFT results (shown in Fig. 1), that the coverage with Si−Me−Fc is about 2 to 3 monolayers, while the coverage corresponds to a single monolayer in the case of Si−UA−Fc. This also accounts for the reduced intensity of Fe 2p structures in Si−UA−Fc compared to Si−Me−Fc.

### Electrochemical studies

**Figure 4:**
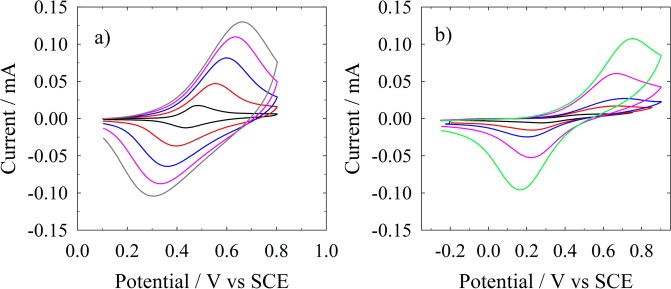
Cyclic voltammetry curves, 0.1 M TBATBF in ACN: (**a**) Si−Me−Fc, scan rate: 10 (black), 50 (red), 100 (blue), 200 (pink), 500 (gray) mV s^−1^ (**b**) Si−UA−Fc, scan rate: 10 (black), 50 (red), 100 (blue), 200 (pink), 500 (gray) mV s^−1^.

Attachment of the redox-active ferrocene moiety and its electrochemical kinetic studies were performed by conventional CV measurements. Figure 4a,b shows CV curves as a function of the scan rate, the latter are recorded by using the Si−Me−Fc and Si−UA−Fc interfaces as the working electrode. In the case of the Si−Me−Fc a one electron oxidation process was observed at +0.45 V, while reduction process occurs at +0.35 V (vs. SCE), compare Fig. 4a. As expected the oxidation process shifted towards more positive potential, while reduction process shifted towards more negative potential upon increasing the scan rates. Indeed, the peak-to-peak CV separation (ΔE_p_) increases with the scan rate (compare Fig. 3SI in the Supporting Information). From a qualitative point of view, the cyclic voltammetry results suggest a quasi-reversible in nature oxidation process of the grafted ferrocene, this because the peak potentials of the forward and backward scan are always shifted by a not-negligible voltage value, while a true reversible process should feature the same (reduction and oxidation) potential values^3^. Using the cyclic voltammetry data, surface coverage of the Si−Me−Fc monolayer was determined at 4.3 × 10^−10^ mol/cm^2^ considering i_p_ vs. ln(ν)^46^. For a short molecule, this surface coverage value indicate that monolayers are reasonably well packed^47–49^. Linear behaviour of the Faradic peak current as a function of the scan rates strongly indicate that Fc−CH_2_−OH successfully covalently grafted on the silicon substrate. The ratio between anodic and cathodic peak current was experimentally found just larger than one (again in agreement with a quasi-reversible behaviour). Figure 4b sets out CVs recorded in the case of the Si−UA−Fc electrode, the overall behaviour is similar to that one of the shorter molecular-spacer with a more prominent less-reversible behaviour: larger ΔE_p_ values and smaller current values with respect to the Si−Me−Fc system. We further investigated full width at half maximum (FWHM) at different scan rates, which were found higher than the ideal Nernstian behaviour of 90.6/n mV. This deviation can be ascribed to stray electrostatic effects induced by adjacent charged species not involved in the redox process^50^.

Table 1 sets out the experimental parameters obtained by treatment of data in the Fig. 4a,b. A substantial large variation is observed in the electron-transfer rate constant, ***K***_***ET***_, experimental values present in the literature relevant to ferrocene moieties grafted on Si single crystal surfaces (ranging between 4500 to 3 s^−1^, such a large variation indicates a high sensitivity of electrochemical measurements to the structure, which is a function of the chemical preparation, of these hybrid interfaces)^25,43,44,47–49,51^. Note that all the elaboration of the experimental electrochemical evidence (details are reported in the Supporting Information) was carried out assuming a one electron single step charge transfer mechanism, allowing for an effective rationalization of the experimental overall picture. Which is the same mechanism underlying the theoretical analysis reported in ref.^34^, comparing the experimental values of charge transfer rate constants with the theoretical ones, we can conclude that the charge transfer dynamics follows a Marcus mechanism in the case of the Si−Me−Fc, while in the case of Si−UA−Fc the agreement is not so good (the experimental charge transfer rate constant is much larger than the theoretical one. Theoretical values obtained by first principle calculations carried out within the Marcus paradigm yield ${k}_{ET}=77.8\,{s}^{-1}$ and ${k}_{{ET}}=1.3\times {10}^{-9}\,{s}^{-1}$ for Si−Me−Fc and Si−UA−Fc, respectively^36^). Experimentally, the Si−Me−Fc electrode shows a more reversible behavior with respect to the Si−UA−Fc one, as can be inferred by i) standard potential values (the E° of Si−Me−Fc is smaller than the Si−UA−Fc one, compare the data in Table 1) ii) the peak-to-peak potential separation (ΔEp) is smaller for Si−Me−Fc than for the Si−UA−Fc: for the same value of scan rate, compare for instance the potential peak-to-peak separation in Fig. 4a (Si−Me−Fc, at 500 mV s^−1^ scan rate gray curve) ΔEp = 300 mV, while Fig. 4b (Si−UA−Fc, at 500 mV s^−1^ scan rate gray curve) ΔEp = 600 mV indicating a more reversible behavior of the Si−Me−Fc than for the Si−UA−Fc.

**Table 1:** Standard potential (E°), molecular surface coverage (Γ), ET rate constant (*k*_*ET*_) and charge transfer coefficient (α) As obtained by CV data.

		SiMeFc	SiUAFc
*Laviron* ^47^	*k*_*ET*_/s^−1^	0.84	0.026
	α	0.61	0.69
*CV Simulation* ^b^	E°/V vs SCE	0.46	0.59
	*k*_*ET*_/s^−1^	0.98	0.009
	α	0.45	0.40
	Γ/mol cm^−2^	3.6 × 10^−10^	4.0 × 10^−10^
*i*_*p*_ *vs*. *ln(v)*^48^	Γ/mol cm^−2^	4.3 × 10^−10^	4.7 × 10^−10^

^a^CH Instrument simulation program version 9.24.

### Atomic force microscopy

**Figure 5:**
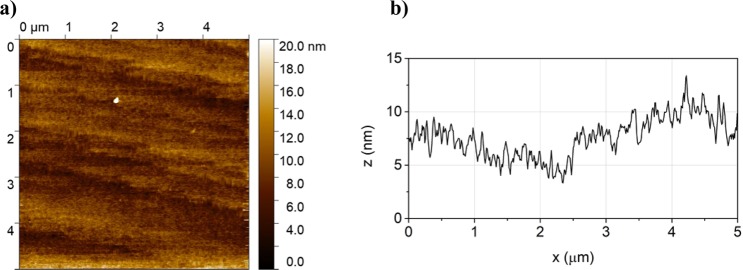
AFM scans of the Si-UA-Fc sample (scan area 5.0 µm × 5.0 µm): (**a**) surface morphology (**b**) heights profile related to the horizontal mid cross section.

To assess the influence of the functionalization process on the morphology of the surface, AFM images were recorded. Figure 5a shows the surface morphology of the Si−UA−Fc hybrid interface, which is the sample subjected to the largest number of electrochemical and chemical treatment steps. Figure 5b sets out the profile (section measured at 2.5 mm height with respect to the vertical axis) in the middle of the scan relevant to Fig. 5a morphology: the sample appears rather flat at the nanoscale, confirming that the functionalization and the sustained treatments did not end up in any dramatic damage of the surface.

For the sake of comparison AFM measurements were performed also on the Si-Me-Fc sample, on the silicon functionalized with the undecanoic acid (Si-UA) and bare Si (roughness values, images and surface profiles are reported in the Supporting Information). The root mean square (RMS) roughness, R_rms_, value was used as indication of the surface coarseness induced by the different treatments. The following R_rms_ values are obtained: Si 1.60 (±0.2) nm, Si-UA 1.49 (±0.15) nm, Si-Me-Fc 0.90 (±0.1) nm, Si-UA-Fc 1.41 (±0.15) nm. The variations of R_rms_ in the different samples are minimal and comparable with the roughness of the bare pristine silicon substrate; indeed, we can conclude that the functionalization treatments do not result in surfaces with higher roughness with respect to the original pristine silicon.

## Conclusions

Redox active ferrocene was covalently immobilized onto the silicon surface via SiOC and SiC covalent bonds. From the experimental point of view, our electrochemical experimental results are in line with the evidence reported by Simonet^52^, Zannoni *et al*.^41,49^ and Calborean^21^, when dealing with similar hybrid interfaces. The striking result is that the connection of the iron redox couple via a chain of single (N.B: not conjugated) covalent bonds (even in the case of a long, in principle not conducting, alkyl chain) acts as an on/off switch on the ET process involving the ferrocene: the charge of the interface is driven by the external applied potential (bias), which can be switched on (charged, i.e. oxidized ferrocene) and off (discharged, i.e. neutral ferrocene) following the application of a suitable value of the bias. Moreover, the results here reported show that the efficiency of the on/off (charge/discharge) process is a function of the structure of the linker placed between the redox centre (ferrocene) and the electrode surface (in this case silicon). This result is at variance with respect to the case of an alkyl SAM functionalized electrode surface (1decanethiol on Au), but featuring the ferrocene in bulk solution: in this case the Faradaic current is almost zero although the distance between the ferrocene and the electrode surface, as well as the chemical nature of the spacer, are quite similar^4^. Moreover, a semi-quantitative agreement between the experimental and theoretical values is found for the “short spacer” ferrocene derivative, also XPS data reveals well-defined signals including elemental compositions. For the Si−UA−Fc system a much larger, than theoretically expected, experimental *K*_*ET*_ value is found. A result in line with findings recently discussed in the literature, where high current values are found (i.e. CVs featuring evident redox current peaks) even dealing with long saturated alkyl-carbon chains, where a non-conducting behaviour is expected^3,20,22,28^. Compare the results discussed in A. Nitzan, *Chemical dynamics in condensed phases relaxation*, *transfer and reactions in condensed molecular systems*: page 600 Figure 16.9. Eventually, the experimental charge transfer rate constant for the Si-Me-Fc electrode, *K*_*ET*_ = 0.98 s^−1^, is found to be smaller than the theoretical value of 77.8 s^−1^, the latter was obtained by application of the Marcus theory, which represents a theoretical upper ideal limit.

As a whole, it must be noted that surface engineering with ferrocene immobilized moieties was successfully achieved on H-terminated silicon substrate, a route which is promising in building and tailoring nanoscale devices for applications in the field of molecular electronics, spintronics, photonics, catalytic properties.

## Materials and Methods

### Materials and Reagents

All the reagents used in this work are from Sigma, unless otherwise stated (compare the synthetic details in the Supporting Information), and used as received without any further purification. Highly n-doped silicon substrates (degenerate) with a resistivity <0.010 ohm.cm and thickness in the 475 to 525 µm range, were purchased from Siltronix.

### Preparation and functionalization of H-terminated Si(111) surface

Silicon substrates were cleaned by sonication in n-hexane, acetone, then ethanol (15 min each) and then, dried under a N_2_ stream. Hydrogen terminated Si(111) surfaces [H-Si(111)] were prepared following the procedure described by Dumas and Kato^53,54^. A series of acid and basic hot bath cleaning steps were performed followed by a final NH_4_F treatment^54^. Functionalization of the H-terminated Si(111) surface was carried out using a standard Schlenk-line procedure for reaction in inert atmosphere. Photo-induced grafting of was carried out by covering the H-terminated Si(111) surface with Fc-CH_2_OH and irradiating the sample for 1 h with a power of ~35 mW cm^−2^ using a quartz–iodine lamp with the main emission peak at 236 nm. The flask was heated above the melting point of Fc-CH_2_OH, at 56 °C.

### Electrochemical measurements

Cyclic voltammetry (CV) measurements were performed using CHI 660 A and Metrohm Autolab Pgstat 128 N potentiostats, in a typical three-electrode electrochemical cell arrangement. Fc-CH_2_-OH modified H-terminated Si(111) substrates used as a working electrode (active area = 78 mm^2^), a Pt wire and a Saturated Calomel Electrode *Hg/Hg*_2_*Cl*_2_*/KCl*_*sat*_ (SCE) electrodes used as the counter and reference electrodes, respectively. Ohmic contacts were made by rubbing indium-gallium (In-Ga) eutectic alloy (495425 Aldrich) on the back of the previously scratched to further improve the contact^51^. 0.1 M tetrabutylammonium tetrafluoroborate (TBATFB) in acetonitrile (ACN) was the base electrolyte.

### X-ray photoelectron spectroscopy (XPS) measurements

For XPS measurements, Mg K_α_ radiation was used from a dual anode non-monochromatic X-ray source (VG-XR3) operated at 15 mA, 15 kV. Spectra were acquired with double-pass cylindrical mirror analyzer (PHI-15-255G) at constant pass energy of 50 eV.

### Atomic force microscopy (AFM) measurements

Atomic force microscopy (AFM) (PicoSPM, Molecular Imaging, Tempe, AZ, USA) was used to acquire images of the samples (512 px × 512 px, 5 µm × 5 µm) and evaluate the roughness. The measurements were performed in contact mode with a non-conductive Si_3_N_4_ triangular cantilever (NP-S10, Veeco, Plainview, NY, USA) with the following features: 0.4–0.7 μm range in depth, 0.12 N/m force constant, 0.5 V force set point and 1.21 l/s speed.

## Supplementary information

Supporting Information
