# Supplementary material for: Redox-Active Ferrocene grafted on H-Terminated Si(111): Electrochemical Characterization of the Charge Transport Mechanism and Dynamics
Source: Sci Rep. 2019 Jun 19;9:8735. doi: 10.1038/s41598-019-45448-w (PMC6584626; doi:10.1038/s41598-019-45448-w)
Supplement: Supplementary file 1 — Supporting Information [file 41598_2019_45448_MOESM1_ESM.pdf]

# Supporting Information

## *Redox-Active Ferrocene grafted on H-Terminated Si(111): Electrochemical Characterization of the Charge Transport Mechanism and Dynamics.*

**Claudio Fontanesi [a,b*], Enrico Da Como[b], Davide Vanossi[c], Monica Montecchi[a], Maria Cannio[a], Prakash Chandra Mondal[d], Walter Giurlani[e], Massimo Innocenti[e], Luca Pasquali[a,f,g]**

[a]DIEF, Univ. of Modena and Reggio Emilia, via Vivarelli 10, 41125 Modena, Italy.

[b]Dept. of Physics, Univ. of Bath, Claverton Down, Bath BA2 7AY, United Kingdom.

[c]DSCG, Univ. of Modena and Reggio Emilia, via Campi 183, 41125 Modena, Italy.

[d]Department of Chemistry, Indian Institute of Technology, Kanpur 208016, India.

[e]Dept. of Chemistry, Univ. of Firenze, via della Lastruccia 3, 50019 Sesto Fiorentino, FI, Italy.

[f]IOM-CNR Institute, Area Science Park, SS 14 Km, 163.5, Basovizza, 34149 Trieste, Italy

[g]Department of Physics, Univ. of Johannesburg, P.O. Box 524, Auckland Park 2006, South Africa

**1 Experimental**

*1.1 Synthesis of organic intermediates.*

*1.1.1 Synthesis of N,N,N',N'-tetramethyldiaminomethane*

An aqueous solution of dimethylamine 25% (52 mL,15.86 g, 0.352 mol) was added to an ice cold stirred solution of aqueous formaldehyde 37% (13 mL, 4.81g, 0.176 mol), maintaining the bath temperature below 15 °C. After 30 min of stirring solid KOH was added until the formation of two separated phases. The upper phase was removed and dried over solid KOH. After filtration the crude product was distilled obtaining 5.64 g (65% of yield) of N,N,N',N'-tetramethyldiaminomethane (bp: 82-84 °C).

$^1$H NMR (200 MHz, $CDCl_3$): δ = 2.48 ppm (12H, s, $CH_3$), δ = 2.80ppm (2H, s, $CH_2$)

*1.1.2 Synthesis of Dimethylaminomethylferrocene.*

A mixture of N,N,N',N'-tetramethyldiaminomethane (2.800 g, 27.4 mmol), paraformaldehyde (0.823 g, 27.4 mmol) and 20.7mL of glacial acetic acid was heated until complete dissolution of the solid phase. Then, ferrocene was added (10.210 g, 54.8 mmol) and the mixture was refluxed for 5h. The solution was cooled with 60 mL of water and the resulting mixture was filtered. The recovered solid was washed with diluted acetic acid and water (this solid is not-reacted ferrocene that can be

* author to whom correspondence should be addressed

used for another aminomethylation reaction). The liquid phase was cooled with an ice bath and made strongly alkaline with a 50% w/w NaOH solution. The resulting mixture was extracted three times with diethyl ether (20 ml) and the combined organic phase was washed with water (40mL), dried with $MgSO_4$ and filtrated. After evaporation of the solvent 5.601g of a dark oil (42% of yield) of pure N.N-dimethylaminomethylferrocene were obtained.

$^1$H NMR (200 MHz, $CDCl_3$): δ = 2.18ppm (6H, s, $CH_3$), δ = 2.60 ppm (2H, s, $CH_2$), δ = 4.10 ppm (7H s $C_5H_4$) 4.17 ppm (2H, d, $C_5H_4$)

*1.1.3 Methylation of N.N-dimethylaminomethylferrocene*

A solution of methyliodide (2.52 mL. 5.74 g, 40.7 mmol) in 4 mL of methanol was added dropwise to a cold solution of N.N-dimethylaminomethylferrocene (6.537 g, 27.1mmol) in 10 mL of methanol. The mixture was heated and refluxed for 15 min then 50 mL of diethyl ether were added. The resulting precipitate was collected and washed with diethyl ether until the washing solvent was colourless and then dried under vacuum. Finally, 6.859 g (66% of yield) of the quaternary ammonium salt were obtained in the form a yellow powder.

$^1$H NMR (200 MHz, $CD_3CN$): δ=2.19ppm (2H, s, $C_5H_4CH_2$-), δ=2.98ppm (9H, s, -N$CH_3$), δ=4.28ppm (5H s $C_5H_4$), 4.40ppm (2H, m, $C_5H_4$), 4.52ppm (2H, m, $C_5H_4$)

*1.1.4 Synthesis of Hydroxymethilferrocene* (MeFc)

The quaternary ammonium salt of ferrocene (6.859 g, 17.8 mmol) was added to a solution of NaOH 1M (70 mL) and the mixture was heated to reflux for 3 h. Then the reaction mixture was cooled to room temperature and extracted 3 times with 30 mL of diethyl ether. The combined organic phase was washed once with 30 mL of water and dried with MgSO4. After filtration the solvent was evaporated and the product recrystallized from hexane obtaining 3.443 g (90% of yield) of hydroxymethilferrocene (MeFc), Chart 1SI.

$^1$H NMR (200 MHz, $CD_3CN$), δ = 4.16 ppm (2H s $C_5H_5$*$CH_2$*-) δ = 4.18 ppm (5H, s, $C_5H_5$), δ = 4.23 ppm (2H t, $C_5H_5$), δ=4.32 (2H s $C_5H_5$).

**CHART 1SI**

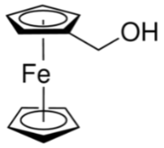


*1.1.6 Synthesis of 11-Iodoundecanoic acid*

A mixture of 3 g of 11-bromoundecanoic acid (11.3 mmol), 50 mL of acetone and 5.67 g of KI was heated to reflux for 6 h. After filtration, and subsequent solvent evaporation, the solid part was extracted by diethyl ether. After evaporation of the solvent, 2.87 g (85% of yield) of 11-iodoundecanoic acid were obtained, in the form of a pale yellow powder.

NMR (200 MHz, DMSO d6), δ = 3.25 ppm (2H, s, I$CH_2$-), δ = 1.75ppm (2H, s, I $CH_2CH_2$-), δ = 1.50 ppm (2H, s, HOOC$CH_2CH_2$-), δ = 2.18 ppm (2H m, HOOC$CH_2$-), δ = 11.90 ppm (1H s,COOH).

*1.2 Si(111) surface functionalization*

Two different molecular architectures grafted on the Si(111) surface were designed and synthetized, at variance with typical functionalization obtained by thiol chemisorption of gold or valve metals[1].

In the first one, the ferrocene (Fc) moiety was covalently grafted through a Si – O covalent bond to the Si(111) surface *via* a –O–$CH_2$– molecular spacer (in the following addressed as Si–Me–Fc).

In the second one, the Fc moiety was covalently grafted through a Si – C covalent bond to the Si(111) surface *via* a much longer alkyl chain, –$(CH_2)_{10}$–COO–$CH_2$– , molecular spacer (addressed as Si–UA–Fc). The functionalization of the H-terminated Si(111) surfaces was carried out following the procedure described by Dumas and Kato[2,3]. Functionalization of the H-terminated Si(111) surface was carried out using a standard Schlenk-line procedure for reaction in inert atmosphere. For the preparation of Si–Me–Fc, photo-induced grafting of MeFc was carried out by covering the H-terminated Si(111) surface with MeFc and irradiating the sample for 1 h with an estimated power of about 35 mW $cm^{-2}$ with a quartz–iodine lamp that had the emission peak at 236 nm. The flask was heated above the melting point of MeFc, at T = 329 ± 1 K (Scheme 1SI). Alternatively, the functionalization was achieved through a thermal induced reaction by heating (in the dark) at a higher temperature: T = 360 ± 1 K.

**Scheme 1SI**

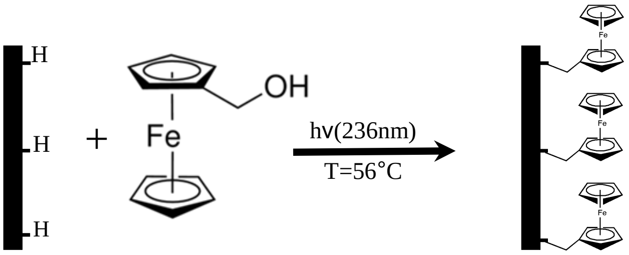


For the preparation of Si–UA–Fc, the ferrocene group was chemi-adsorbed on the surface through a long chain aliphatic spacer. The H-Si(111) surface was first functionalized by electrochemical assisted grafting of the –$(CH_2)_{10}$–COOH moiety. This was achieved by using the Si(111) as the working electrode in an electrochemical cell, where a potential of –1.7 V vs SCE was applied for 10 minutes to a 1 mM acetonitrile solution of 1–iodoundecanoic acid, containing 0.1 M NaBF4 as the support electrolyte. At this potential the 11-iodoundecanoic acid dissociates to yield iodide and the neutral open shell radical through the following reaction[4,5]:

$$I(CH_2)_{10}COOH + e^- \longrightarrow (CH_2)_{10}COOH^\bullet + I^-$$

The radical then attacks the Si-H surface, and the total process can be represented as:

$$Si(111)_{H-terminated} + I(CH_2)_{10}COOH + e^- \longrightarrow Si(111) - (CH_2)_{10}COOH + I^-$$

This surface was then post-functionalized: the grafted undecanoic acid was transformed in the acyl halide via reaction with $SOCl_2$, and the halide was finally made to react by mild heating in an hexane solution of MeFc, to give the $Si(111) - (CH_2)_{10} - COO - CH_2 - FC$ hybrid interface (Scheme 2SI).

**Scheme 2SI**

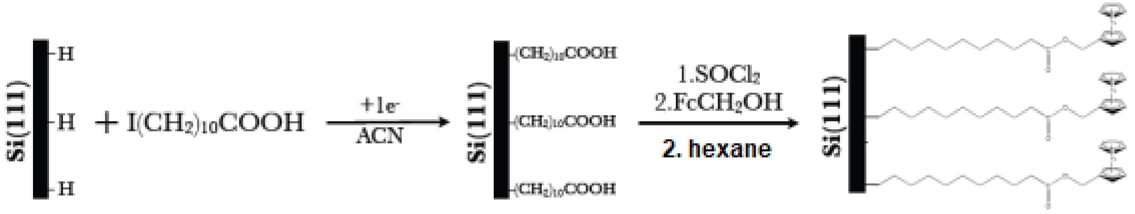


## 2 Laser Ablation Inductively Coupled Plasma Mass Spectrometry (LA-ICP-MS).

ICP was performed with a New Wave laser ablation UP 213 unit, equipped with a solid state laser - exciting wavelength of 213 nm, energy density > 27 J/cm$^2$, tuneable pulse repetition rate in the 1 to 20 Hz frequency - followed by a plasma spectrometer HR-MC-ICPMS Neptune, by Thermo Fisher Scientific.

### *2.1 LA–ICP–MS results*

LA–ICP–MS spectra ($^{56}$Fe signal) were recorded aiming to probe (semi-quantitatively) the surface concentration and distribution of the Fc redox probe. Figure 1SI shows the LA–ICP–MS $^{56}$Fe mass signal recorded for both the Si–Me–Fc and Si–UA–Fc surfaces. The signal intensity is roughly of the same intensity and independent of the position of the sampled surface zone. This suggests that the functionalization procedures here exploited are able to yield a rather uniform functionalized surface coverage for both the Si–Me–Fc and Si–UA–Fc surfaces (similar cps counts in Figure 1a and b SI, with a slightly larger signal, higher coverage, for the Si–Me–Fc surface).

**Figure 1SI**

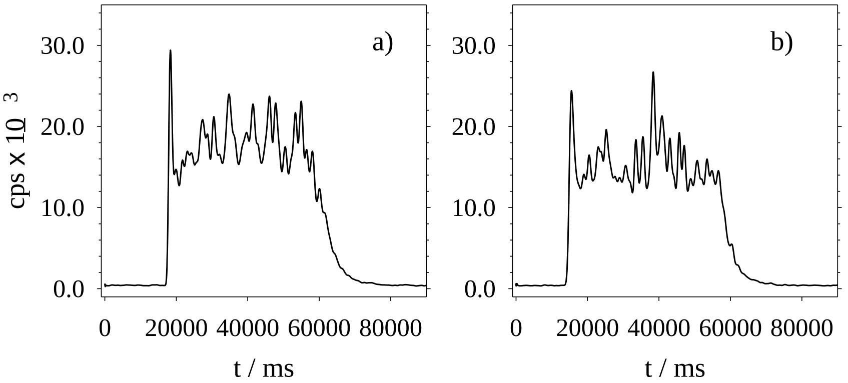


**Figure 1SI**. Cps/time $^{56}$Fe mass spectra are reported: a) Si–Me–Fc interface b) Si–UA–Fc interface.

## 3 XPS additional information

Here we report spectra as a function of binding energy, using as a reference the C 1s main peak position at 284.8 eV.

**Figure 2SI**

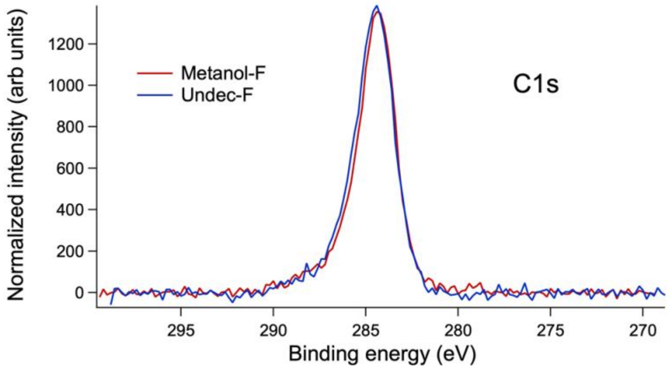


**Figure 2SI.** C1s acquired on Si–Me–Fc and Si–UA–Fc surfaces.

Figure 2SI shows the detail of XPS spectrum, C1s detail. No traces of I 3d could be observed by XPS. Please note that I3d spectra would be superimposed to Auger of Fe. I3d is not distinguishable at about 620-630 eV. See below the Auger spectrum: Figure3SI.

**Figure 3SI**

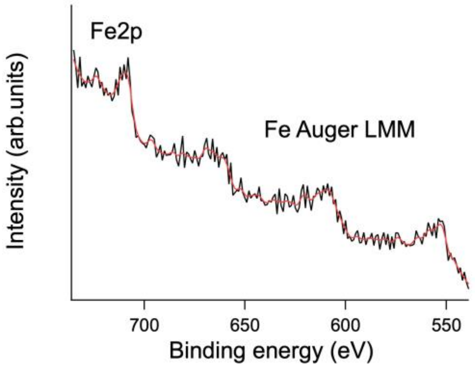


**Figure 3SI.** Si–UA–Fc surfaces: region of Fe Auger and Fe2p in wide scans.

## 4 Analysis of CVs data

Figure 4SI sets out the Laviron[6] plot, which reports the anodic and cathodic peak potentials as a function of the logarithm of the scan rates i.e., Ep vs ln(v), relevant to the CV data shown in Figure 4a,b of the main manuscript. Interpolation of the experimental data in Figure 4SI, in the 0.01 to 1.0 $Vs^{-1}$ scan rate range, allows to estimate the values of the charge transfer rate constant, $k_{ET}$, and of the (apparent) charge transfer coefficient ($\alpha$)[6,7]; the relevant kinetic values are listed in Table I of the main manuscript. The quasi-reversible nature of the redox process is also corroborated by the

comparison of the amount of exchanged charge relevant to the oxidation and reduction faradaic processes (the integral under the current peaks in the forward and backward scans); almost the same value of charge is found for the forward and backward scans (± 5%). The ferrocene surface coverage can be thus estimated exploiting the dependence between the peak current (CV curves) and the scan rate[8]: $i_p = n^2F^2\nu A\Gamma/4RT$, where: $\boldsymbol{n}$ is the number of exchanged electrons (one in the present case), $\boldsymbol{F}$ is the Faraday, $\boldsymbol{A}$ the area of the electrode surface, $\boldsymbol{\Gamma}$ is the surface concentration. The relevant experimental values are reported in Table I main manuscript. The CVs in Figures 4a and 4b (compare the main manuscript) are fitted by using the CHI simulation program Version 9.24.

**Figure 4SI**

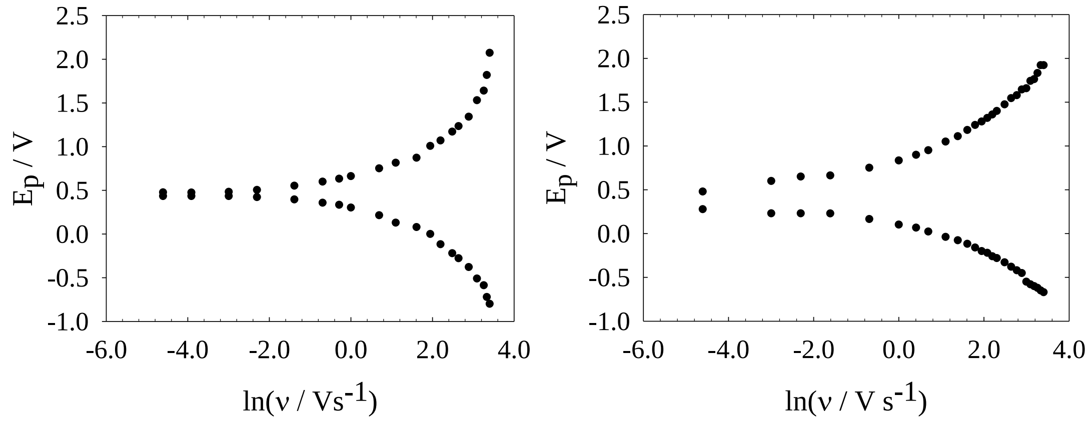


**Figure 4SI.** *Left*: Ep vs ln(v) plot referring to CV shown in Figure 3a of the main manuscript. *Right*: Ep vs ln(v) plot referring to CV shown in Figure 3b of the main manuscript. Data are shown up to 20 V s$^{-1}$ scan rate.

The parameters of the fit are reported in Table I main manuscript. The fitting of each CV curve yields also an estimate of the surface concentration of the ferrocene redox couple (differently from the Laviron approach[6], whose surface concentration value stems as averaged from a family of CV curves recorded at various potential scan rates). Note that, the parameters relevant to the characterization of the electron transfer dynamics, i.e. $k_{ET}$ and α, although obtained following two procedures which are independent in nature (the fitting of the CVs, at each scan rate value, and the Laviron treatment), turn out as being in rather good agreement. Please compare Table I of the main manuscript.

***5 AFM additional information.***

Figure 5SI

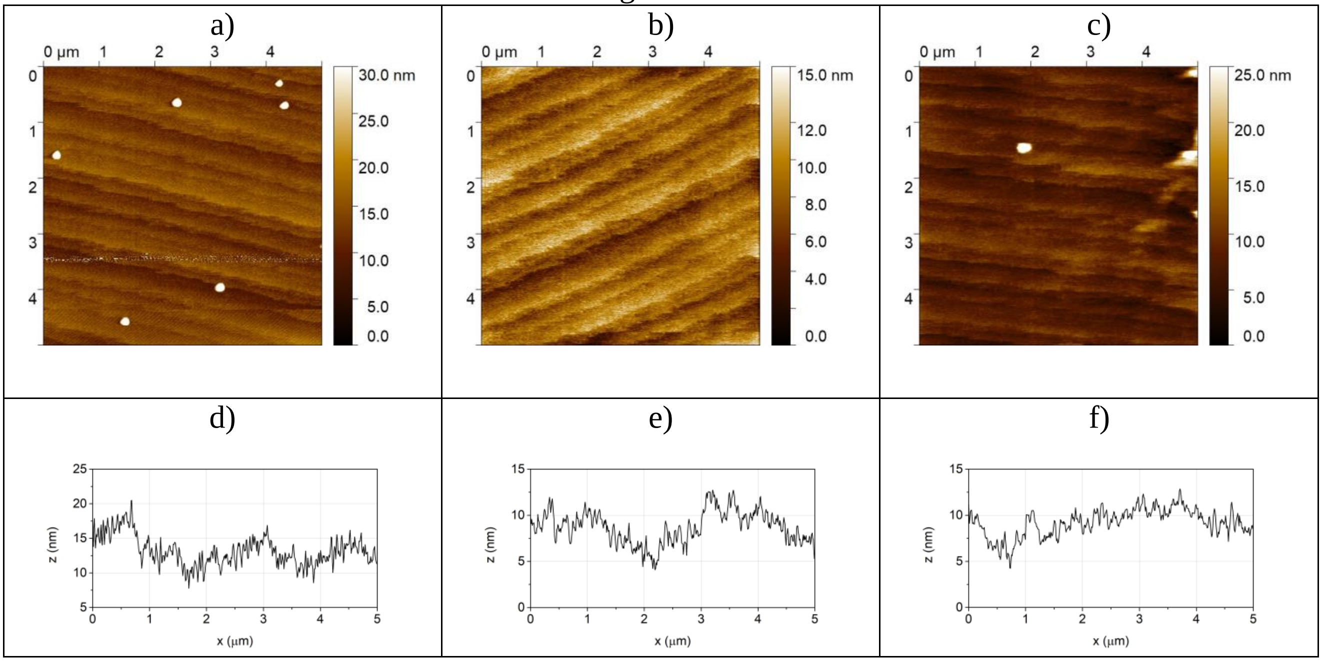


Figure 5SI. AFM scans: a) and d) the pristine silicon; b) and e) Si-UA; c) and d) Si-UA-Fc. Sscan area 5.0 µm × 5.0 µm. Upper figures surface morphology. Lower figures heights profile related to the horizontal mid cross section

**Table 1SI.** Roughness value as obtained from the elaboration of the AFM images.

| **Silicon** | Si–UA | Si–UA–Fc | Si–Me–Fc |
|---|---|---|---|
| Average value: 7.9648 nm | Average value: 6.8615 nm | Average value: 4.32238 nm | Average value: 5.5970 nm |
| RMS roughness (Sq): 1.59482 nm | RMS roughness (Sq): 1.48739 nm | RMS roughness (Sq): 875.374 pm | RMS roughness (Sq): 1.40772 nm |
| Mean roughness (Sa): 1.26745 nm | Mean roughness (Sa): 1.17714 nm | Mean roughness (Sa): 697.594 pm | Mean roughness (Sa): 1.11596 nm |
| Minimum: 0.0000 nm | Minimum: 0.0000 nm | Minimum: 0.00000 nm | Minimum: 0.0000 nm |
| Maximum: 20.9509 nm | Maximum: 14.7664 nm | Maximum: 8.48962 nm | Maximum: 16.7042 nm |
| Median: 7.9484 nm | Median: 6.8618 nm | Median: 4.32164 nm | Median: 5.5828 nm |

## 6 Theoretical, DFT, cluster geometry

**Figure SI**

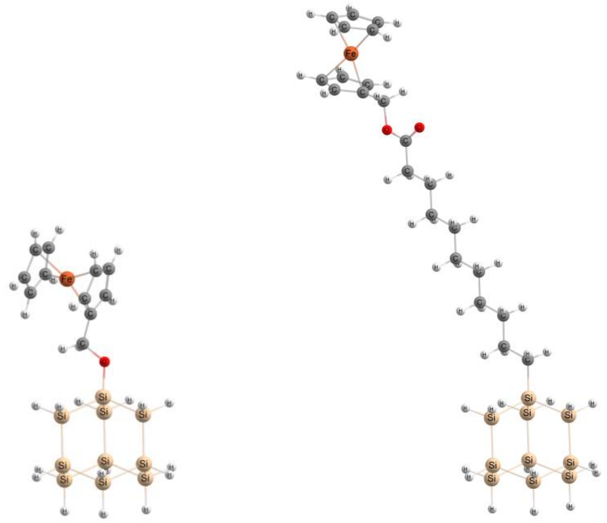

**Figure SI.** DFT Optimized structures: left Si–Me–Fc and right Si–UA–Fc surfaces

Si–Me–Fc: optimized coordinates, pbe1pbe/6-31+g* level of the theory.

| | | | |
|---|---|---|---|
| Si | -0.008454000 | 0.474817000 | 0.129560000 |
| Si | -0.153350000 | -0.554551000 | 4.528293000 |
| Si | 1.871856000 | -0.090552000 | 3.433672000 |
| Si | -0.764652000 | -3.494895000 | 2.092452000 |
| Si | -0.635612000 | -2.861346000 | 4.352900000 |
| Si | 1.722628000 | -0.720777000 | 1.172990000 |
| Si | -1.905714000 | 0.621702000 | 3.466743000 |
| Si | -2.045267000 | 0.005880000 | 1.201510000 |
| Si | 1.275598000 | -3.022026000 | 1.029877000 |
| Si | -2.491294000 | -2.294242000 | 1.047938000 |
| H | -1.672039000 | 2.093952000 | 3.570368000 |
| H | -3.213476000 | 0.328655000 | 4.130558000 |
| H | 2.174438000 | 1.368101000 | 3.528073000 |
| H | 2.982088000 | -0.843487000 | 4.088589000 |
| H | 0.423889000 | -3.663014000 | 5.037408000 |
| H | -1.936057000 | -3.177864000 | 5.020538000 |
| H | -3.147680000 | 0.778258000 | 0.547844000 |
| H | 3.020163000 | -0.420758000 | 0.490911000 |
| H | 1.209697000 | -3.441431000 | -0.402113000 |
| H | -3.794316000 | -2.603973000 | 1.709085000 |
| H | -2.598159000 | -2.698587000 | -0.385773000 |
| H | -1.049097000 | -4.961610000 | 2.008812000 |
| H | -0.097029000 | 0.088809000 | -1.310576000 |
| H | 0.273382000 | 1.939995000 | 0.193812000 |
| H | 2.374578000 | -3.794507000 | 1.682597000 |
| O | 0.064826000 | -0.073112000 | 6.121805000 |
| C | -0.915053000 | -0.281098000 | 7.135720000 |
| Fe | -1.367637000 | 0.198382000 | 10.222058000 |
| C | 0.642888000 | 0.442294000 | 10.477075000 |
| C | 0.377969000 | -0.437287000 | 9.390691000 |
| C | -0.414432000 | 0.263210000 | 8.429559000 |

| | | | |
|---|---|---|---|
| C | -0.625291000 | 1.585203000 | 8.929420000 |
| C | 0.021617000 | 1.693810000 | 10.191884000 |
| H | -1.208708000 | 2.356850000 | 8.440991000 |
| H | 0.011889000 | 2.561387000 | 10.840161000 |
| H | 1.186979000 | 0.193596000 | 11.379935000 |
| H | 0.691633000 | -1.471694000 | 9.313635000 |
| H | -1.486485000 | -0.814057000 | 12.833406000 |
| H | -2.659476000 | 1.557295000 | 12.306133000 |
| C | -2.360340000 | -1.459812000 | 10.874389000 |
| C | -2.088084000 | -0.581380000 | 11.963360000 |
| C | -2.707887000 | 0.671542000 | 11.684745000 |
| C | -3.363682000 | 0.568207000 | 10.423451000 |
| C | -3.149250000 | -0.749353000 | 9.922891000 |
| H | -2.003443000 | -2.477376000 | 10.772972000 |
| H | -3.500432000 | -1.134301000 | 8.972864000 |
| H | -3.902687000 | 1.361074000 | 9.919544000 |
| H | -1.854501000 | 0.218423000 | 6.857670000 |
| H | -1.123794000 | -1.356103000 | 7.236456000 |

Si−UA−Fc: optimized coordinates, pbe1pbe/6-31+g* level of the theory.

| | | | |
|---|---|---|---|
| C | 9.516541000 | -0.374497000 | 0.133493000 |
| Fe | 12.535034000 | 0.442491000 | -0.045353000 |
| C | 12.016683000 | 2.250787000 | -0.838885000 |
| C | 11.056197000 | 1.258207000 | -1.179685000 |
| C | 10.524588000 | 0.714095000 | 0.030857000 |
| C | 11.160485000 | 1.388493000 | 1.119513000 |
| C | 12.081893000 | 2.330920000 | 0.583201000 |
| H | 10.991905000 | 1.185854000 | 2.170623000 |
| H | 12.744592000 | 2.969469000 | 1.154286000 |
| H | 12.620714000 | 2.817911000 | -1.536428000 |
| H | 10.793826000 | 0.938654000 | -2.181348000 |
| H | 14.908622000 | 0.566093000 | -1.532404000 |
| H | 15.050808000 | 0.736816000 | 1.156791000 |
| C | 13.392418000 | -1.038927000 | -1.153932000 |
| C | 14.355888000 | -0.041096000 | -0.826075000 |
| C | 14.431208000 | 0.049039000 | 0.594530000 |
| C | 13.514616000 | -0.893422000 | 1.144999000 |
| C | 12.872255000 | -1.565562000 | 0.064346000 |
| H | 13.085877000 | -1.324228000 | -2.152773000 |
| H | 12.103852000 | -2.324190000 | 0.153564000 |
| H | 13.316983000 | -1.049280000 | 2.198421000 |
| H | 9.633076000 | -0.947262000 | 1.058900000 |
| H | 9.591624000 | -1.072915000 | -0.705678000 |
| Si | -11.138425000 | 0.491631000 | -2.018560000 |
| Si | -7.200656000 | -0.800804000 | -0.132521000 |
| Si | -8.941105000 | -2.311361000 | -0.611700000 |
| Si | -9.659121000 | 1.116557000 | 2.180767000 |
| Si | -7.565391000 | 0.060486000 | 2.027931000 |
| Si | -11.045977000 | -1.277514000 | -0.476311000 |
| Si | -7.348539000 | 0.983309000 | -1.662172000 |

| | | | |
|---|---|---|---|
| Si | -9.441519000 | 2.042497000 | -1.537794000 |
| Si | -11.356495000 | -0.431851000 | 1.692384000 |
| Si | -9.753107000 | 2.881349000 | 0.633859000 |
| H | -7.127538000 | 0.474872000 | -3.051243000 |
| H | -6.278912000 | 1.990683000 | -1.386503000 |
| H | -8.750082000 | -2.870419000 | -1.985551000 |
| H | -8.886774000 | -3.459928000 | 0.344167000 |
| H | -7.494325000 | -1.048891000 | 3.028540000 |
| H | -6.496801000 | 1.043731000 | 2.384130000 |
| H | -9.494544000 | 3.167484000 | -2.523365000 |
| H | -12.118472000 | -2.273329000 | -0.788825000 |
| H | -12.692237000 | 0.229031000 | 1.793819000 |
| H | -8.694913000 | 3.887634000 | 0.948192000 |
| H | -11.075872000 | 3.569074000 | 0.727689000 |
| H | -9.850688000 | 1.649903000 | 3.565900000 |
| H | -12.472774000 | 1.159378000 | -1.948941000 |
| H | -10.965512000 | -0.035057000 | -3.405641000 |
| H | -11.325213000 | -1.549027000 | 2.683415000 |
| C | -5.507676000 | -1.677918000 | -0.262775000 |
| C | -4.297465000 | -0.755661000 | -0.097206000 |
| C | -2.963319000 | -1.498091000 | -0.147450000 |
| C | -1.757924000 | -0.569861000 | -0.026984000 |
| C | -0.418416000 | -1.299205000 | -0.069061000 |
| C | 0.782787000 | -0.363399000 | 0.031901000 |
| C | 2.124032000 | -1.089757000 | 0.001905000 |
| C | 3.324672000 | -0.152504000 | 0.094295000 |
| C | 4.660107000 | -0.889450000 | 0.081206000 |
| C | 5.849719000 | 0.056475000 | 0.156744000 |
| C | 7.178542000 | -0.657121000 | 0.166385000 |
| H | -5.467163000 | -2.177932000 | -1.241290000 |
| H | -5.484584000 | -2.477671000 | 0.491557000 |
| H | -4.308840000 | 0.009268000 | -0.886883000 |
| H | -4.369334000 | -0.207501000 | 0.853349000 |
| H | -2.933367000 | -2.248806000 | 0.656264000 |
| H | -2.897664000 | -2.058842000 | -1.091941000 |
| H | -1.791691000 | 0.170695000 | -0.840323000 |
| H | -1.831159000 | 0.004136000 | 0.909059000 |
| H | -0.377508000 | -2.036096000 | 0.747083000 |
| H | -0.349054000 | -1.876628000 | -1.003333000 |
| H | 0.745044000 | 0.362384000 | -0.794555000 |
| H | 0.708298000 | 0.227023000 | 0.957667000 |
| H | 2.164828000 | -1.816517000 | 0.826922000 |
| H | 2.196969000 | -1.678763000 | -0.924679000 |
| H | 3.290634000 | 0.560796000 | -0.743278000 |
| H | 3.245879000 | 0.451967000 | 1.010789000 |
| H | 4.706685000 | -1.599016000 | 0.916811000 |
| H | 4.740121000 | -1.496371000 | -0.830194000 |
| H | 5.851844000 | 0.763516000 | -0.683344000 |
| H | 5.802463000 | 0.679873000 | 1.060804000 |
| O | 7.331688000 | -1.858133000 | 0.212682000 |
| O | 8.199211000 | 0.215410000 | 0.120901000 |

## References


1. Fontanesi, C. *et al.* New One-Step Thiol Functionalization Procedure for Ni by Self-Assembled Monolayers. *Langmuir* **31**, 3546–3552 (2015).
2. Mazzara, C. *et al.* Hydrogen-terminated Si(111) and Si(100) by wet chemical treatment: linear and non-linear infrared spectroscopy. *Surf. Sci.* **427**–**428**, 208–213 (1999).
3. Kato, H. *et al.* Preparation of an Ultraclean and Atomically Controlled Hydrogen-Terminated Si(111)-(1× 1) Surface Revealed by High Resolution Electron Energy Loss Spectroscopy, Atomic Force Microscopy, and Scanning Tunneling Microscopy: Aqueous NH 4 F Etching Process of Si(111). *Jpn. J. Appl. Phys.* **46**, 5701–5705 (2007).
4. Fontanesi, C. Theoretical study of the dissociative process of the 4-chlorotoluene radical anion. *J. Mol. Struct. THEOCHEM* **392**, 87–94 (1997).
5. Fontanesi, C., Baraldi, P. & Marcaccio, M. On the dissociation dynamics of the benzyl chloride radical anion. An ab initio dynamic reaction coordinate analysis study. *J. Mol. Struct. THEOCHEM* **548**, 13–20 (2001).
6. Laviron, E. General expression of the linear potential sweep voltammogram in the case of diffusionless electrochemical systems. *J. Electroanal. Chem. Interfacial Electrochem.* **101**, 19–28 (1979).
7. Srinivasan, S. & Gileadi, E. The potential-sweep method: A theoretical analysis. *Electrochimica Acta* **11**, 321–335 (1966).
8. Eckermann, A. L., Feld, D. J., Shaw, J. A. & Meade, T. J. Electrochemistry of redox-active self-assembled monolayers. *Coord. Chem. Rev.* **254**, 1769–1802 (2010).
